# Supplementary material for: Simultaneous Imaging and Therapy Using Epitope-Specific Anti-Epidermal Growth Factor Receptor (EGFR) Antibody Conjugates
Source: Pharmaceutics. 2022 Sep 10;14(9):1917. doi: 10.3390/pharmaceutics14091917 (PMC9505583; doi:10.3390/pharmaceutics14091917)
Supplement: Supplementary file 1 [file pharmaceutics-14-01917-s001.zip › pharmaceutics-1866622-supplementary.pdf]

Simultaneous Imaging and Therapy Using Epitope-Specific Anti-Epidermal Growth Factor Receptor (EGFR) Antibody Conjugates

Anjong Florence Tikum[1], Anand Krishnan Nambisan[1], Jessica Pougoue Ketchemen[1], Hanan Babeker[1,2], Musharraf N. Khan[1], Emina E. Torlakovic[2], Humphrey Fonge[1,3*]

[1]Department of Medical Imaging, College of Medicine, University of Saskatchewan, Saskatoon, SK, S7N 0W8, Canada

[2]Department of Pathology and Lab. Medicine, College of Medicine, University of Saskatchewan, 107 Wiggins Rd, Saskatoon, SK, S7N 5A2, Canada

[3]Department of Medical Imaging, Royal University Hospital Saskatoon, SK, Saskatoon, SK, S7N 0W8, Canada

*Corresponding Author:

Humphrey Fonge PhD

Phone: +1-306-655-3353

Fax: 306-655-1637

E-mail: humphrey.fonge@usask.ca

Supplementary Figures

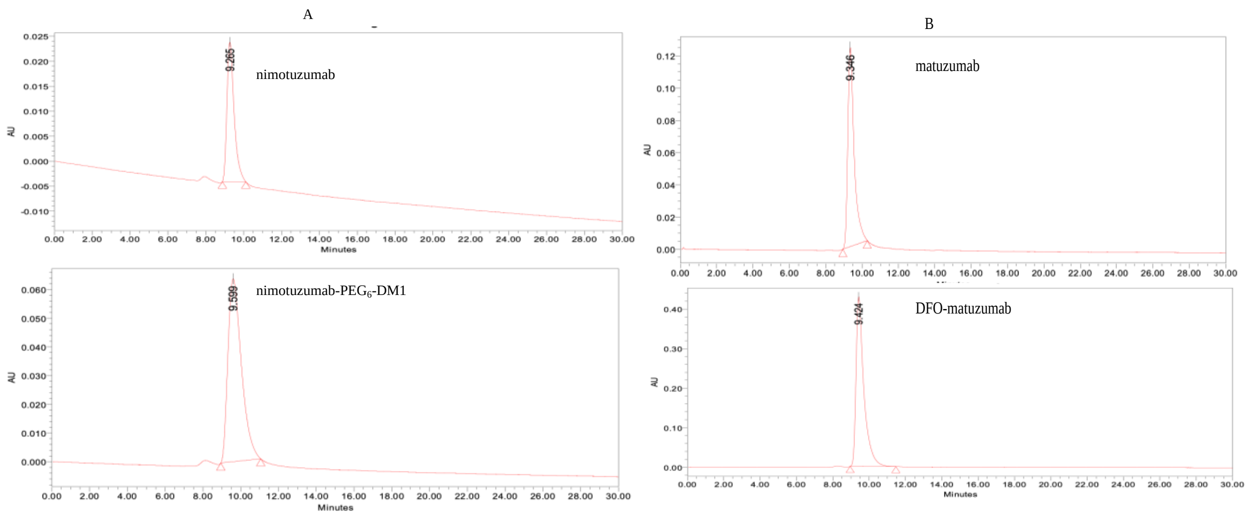

Figure S1: Quality control immunoconjugates using size-exclusion HPLC. A) Nimotuzumab and nimotuzumab-PEG$_6$-DM1, and B) matuzumab and DFO-matuzumab. The purity of all antibodies and immunoconjugates was >= 95% prior to use for *in vitro* and *in vivo* studies

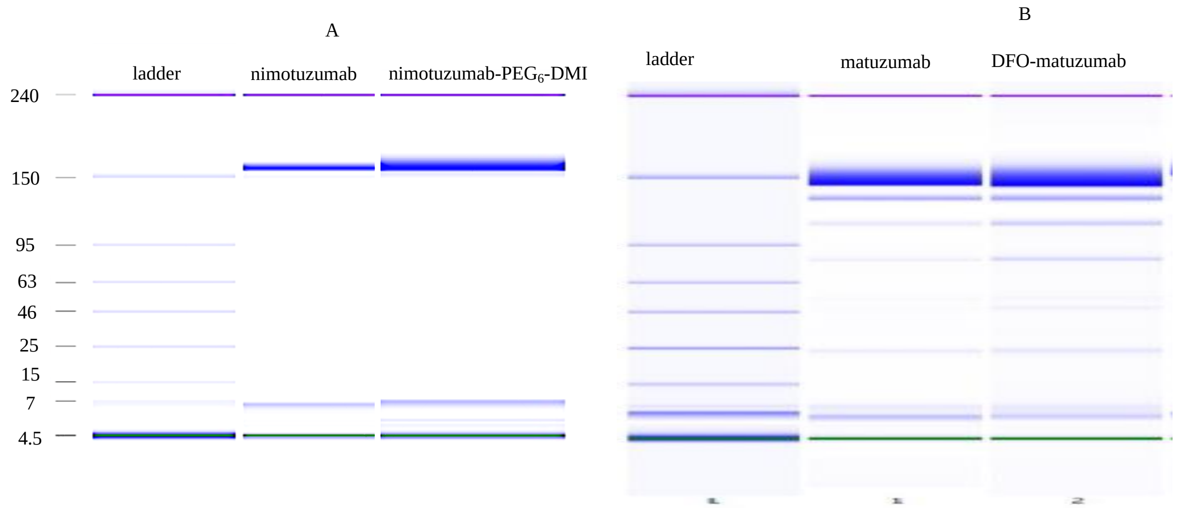

Figure S2: Quality control immunoconjugates using bioanalyzer. A) Nimotuzumab and nimotuzumab-PEG$_6$-DM1 with molecular weights of 155.8, and 161.0 (3.8 drugs) respectively, and B) matuzumab and DFO-matuzumab with molecular weights of 148.1, 151.0 (3.8 DFO) respectively.

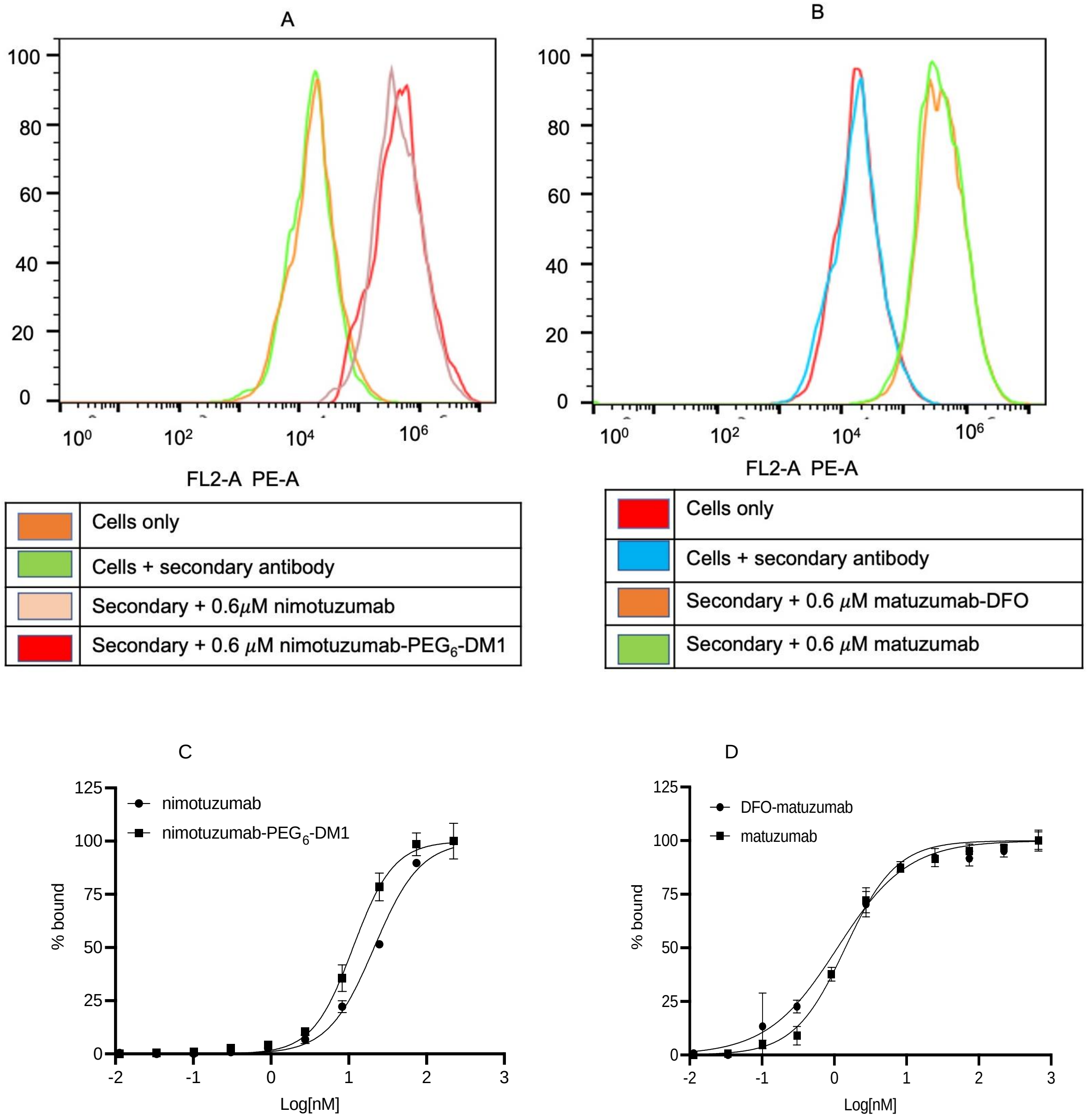

Figure S3: Flow cytometry binding assays. *In vitro* flow cytometry binding assay of SNU-C2B cells following incubation with nimotuzumab and matuzumab conjugates. A) Binding following incubation using 0.6 μM nimotuzumab conjugates, and B) Binding following incubation using 0.6 μM matuzumab conjugates. C) Saturation binding of nimotuzumab and nimotuzumab-PEG_6-DM1, and D) matuzumab and DFO-matuzumab. The estimated K_D values were 5.7 ± 1, 11.4 ± 3, 5.9 ± 1, 6.2 ± 2 nM for nimotuzumab, nimotuzumab-PEG_6-DM1, matuzumab, DFO-matuzumab,

respectively. The binding affinity of the immunoconjugates to the cells were estimated by adding secondary antibody labeled with PE. Cells with secondary antibody and untreated cells were used as control. The mean fluorescent intensity was converted to percentage bound and plotted against concentration and a nonlinear curve fitting was used to estimate the K_D and EC_{50}. All experiments were carried out in duplicates.

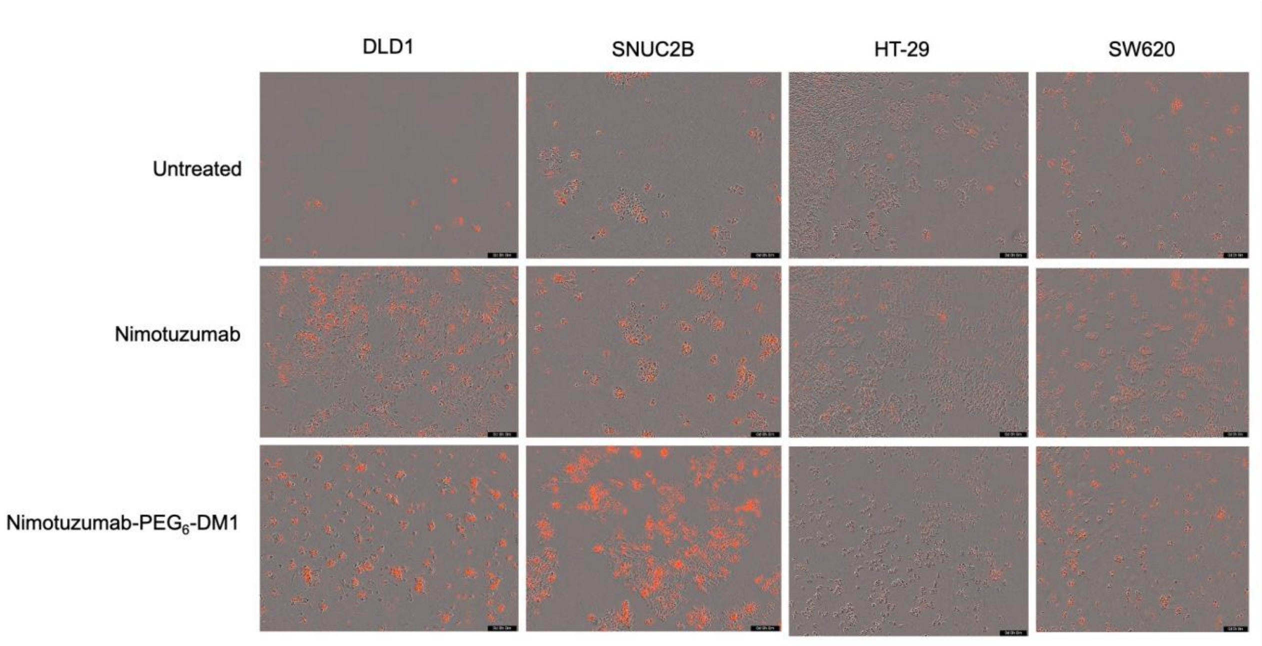

Figure S4: *In vitro* cytotoxicity using Incucyte live cell imaging. Representative phase contrast images of different cells treated with nimotuzumab and nimotuzumab-PEG_6-DM1.
